# Growth of a Novel Nanostructured ZnO Urchin: Control of Cytotoxicity and Dissolution of the ZnO Urchin

**DOI:** 10.1186/s11671-015-1145-3

**Published:** 2015-11-16

**Authors:** Roghayeh Imani, Barbara Drašler, Veno Kononenko, Tea Romih, Kristina Eleršič, Janez Jelenc, Ita Junkar, Maja Remškar, Damjana Drobne, Veronika Kralj-Iglič, Aleš Iglič

**Affiliations:** Laboratory of Biophysics, Faculty of Electrical Engineering, University of Ljubljana, Tržaška 25, Ljubljana, SI-1000 Slovenia; Laboratory of Clinical Biophysics, Faculty of Health Sciences, University of Ljubljana, Ljubljana, SI-1000 Slovenia; Department of Biology, Biotechnical Faculty, University of Ljubljana, Večna pot 111, SI-1000 Ljubljana, Slovenia; Department of Surface Engineering and Optoelectronics, Jožef Stefan Institute, Jamova 39, Ljubljana, SI-1000 Slovenia; Solid State Physics Department, Jožef Stefan Institute, Jamova 39, SI-1000 Ljubljana, Slovenia

**Keywords:** ZnO urchin, Nanowires, Cytotoxicity, Zn ions, Madin-Darby canine kidney, Poly(vinylidene fluorid-co-hexafluoropropylene), 87. Biological and medical physics

## Abstract

The applications of zinc oxide (ZnO) nanowires (NWs) in implantable wireless devices, such as diagnostic nanobiosensors and nanobiogenerators, have recently attracted enormous attention due to their unique properties. However, for these implantable nanodevices, the biocompatibility and the ability to control the behaviour of cells in contact with ZnO NWs are demanded for the success of these implantable devices, but to date, only a few contrasting results from their biocompatibility can be found. There is a need for more research about the biocompatibility of ZnO nanostructures and the adhesion and viability of cells on the surface of ZnO nanostructures. Here, we introduce synthesis of a new nature-inspired nanostructured ZnO urchin, with the dimensions of the ZnO urchin’s acicula being controllable. To examine the biocompatibility and behaviour of cells in contact with the ZnO urchin, the Madin-Darby canine kidney (MDCK) epithelial cell line was chosen as an *in vitro* experimental model. The results of the viability assay indicated that, compared to control, the number of viable cells attached to the surface of the ZnO urchin and its surrounding area were reduced. The measurements of the Zn contents of cell media confirmed ZnO dissolution, which suggests that the ZnO dissolution in cell culture medium could lead to cytotoxicity. A purposeful reduction of ZnO cytotoxicity was achieved by surface coating of the ZnO urchin with poly(vinylidene fluorid-co-hexafluoropropylene) (PVDF-HFP), which changed the material matrix to slow the Zn ion release and consequently reduce the cytotoxicity of the ZnO urchin without reducing its functionality.

**Electronic supplementary material:**

The online version of this article (doi:10.1186/s11671-015-1145-3) contains supplementary material, which is available to authorized users.

## Background

Zinc oxide (ZnO) has been celebrated for the past decade as a photonic material, owing to the wide band gap, high exciton binding energy, and availability of large bulk single crystals [1–4]. Recently, a series of rationally designed nanogenerators (NGs) with piezoelectric ZnO nanowires (NWs) has shown great potential to convert tiny and irregular mechanical energy into electricity [5–7]. The operating mechanism of the electric generator relies on the unique coupling of the piezoelectric and semiconducting properties of ZnO as well as the gating effect of the Schottky barrier formed between the metal tip and the NWs [8, 9]. Based on this mechanism, the NGs constructed from ZnO NWs for harvesting energy from a small-scale dynamic muscle movement, including human finger tapping and the body movement of a hamster, have been developed [10]. This energy could power nanodevices without any battery requirement. The development of wireless nanodevices and nanosystems is of critical importance for bio-sensing, medical science, defence technology and different electronic devices. Furthermore, it is also an essential requirement for implanted biomedical devices, such as biosensors, that they can be self-powered without any battery support. Wireless medical devices may allow *in situ*, real-time biomedical monitoring and detection [11, 12].

The research group of Professor Wang in the USA has applied the AC generator to harvest mechanical energy from body movement *in vitro* and in vivo conditions by implanting the NG in a live rat to harvest energy generated by its breath and heartbeat. This study shows the potential of applying nanogenerators for the scavenging of low-frequency dynamic muscle energy created by a very small-scale physical motion for the possible driving of in vivo nanodevices [13]. For the safe application of NGs in the human body, some crucial problems need to be addressed, such as their biocompatibility and potential cytotoxicity. There have been many reports on the high cytotoxicity and genotoxicity of ZnO nanostructures, which limits their applications in the abovementioned devices in biological systems [14].

It has been reported that the dissolution of Zn ions plays an important role in the cytotoxicity of ZnO [15–18]. The dissolution is strongly increased under acidic conditions as well as in the presence of biological components such as amino acids and peptides [19]. It has been shown that doping with iron [20, 21] or covering the surface of ZnO nanostructures [22] can slow the dissolution of Zn ions and therefore decrease their cytotoxicity.

Herein, we report a growth of nature-inspired ZnO urchins with the carbothermal evaporation route. We hypothesized that the decrease in the Zn ion dissolution could lead to the decrease in the ZnO nanostructure cytotoxicity. In order to decrease the ZnO dissolution in the cell culture medium, the surface of hollow ZnO urchins was covered with a thin layer of poly(vinylidene fluorid-co-hexafluoropropylene) (PVDF-HFP), which is a highly non-reactive polymer with medical and industrial relevance due to its excellent chemical stability and biocompatibility [23, 24]. A thin layer of PVDF-HFP would decrease the Zn ion shedding, resulting in the decrease of the cytotoxicity of ZnO NWs. It has recently been reported as well that the presence of PVDF on the surface of ZnO NWs improves the ZnO NWs’ piezoelectric functionality [25]. In order to assess the biological compatibility of engineered hollow ZnO urchins, the widely used experimental model Madin-Darby canine kidney (MDCK) epithelial cell line was chosen and the cells were cultured on the surface of the hollow ZnO urchins. The aim of this study was to assess the morphological alteration and the ultrastructural status of the MDCK cells grown on the hollow ZnO urchins, which was investigated with scanning electron microscopy (SEM). Furthermore, the neutral red uptake (NRU) assay was carried out for the evaluation of the viability of MDCK cells grown on the hollow ZnO urchins. To evaluate whether the ZnO dissolution happened or not, the Zn content of the cell medium was quantified during the procedure of the NRU assay.

## Methods

### Synthesis of Nanostructured ZnO Urchins

Zinc oxide (ZnO) powder, graphite (C) powder, tin(IV) oxide (SnO_2_) nanopowder with particle size <100 nm, poly(vinylidene fluoride-co-hexafluoropropylene) solution and DMF(N,N-dimethylformamide) were purchased from Sigma-Aldrich (Steinheim, Germany).

The experimental setup for the synthesis of hollow ZnO urchins includes a horizontal tube furnace and a gas supply system as reported previously [26]. A mixture of commercial C: ZnO: SnO_2_ powders in a weight ratio of 1:1:0, 2:1:1 and 3:2:1 was ground and then used as the source material, located in a quartz boat and positioned at the centre of the quartz tube. The P-type silicon (111) wafer covered with gold nanoparticles (NPs) was used as a substrate (Si + Au) and was located downstream of the source materials, at the position where the temperature was 700 °C during the deposition. Thermal evaporation was conducted at 1000 °C for 30 min under atmospheric and nitrogen gas with a flow of 2 L/min, then the tube was kept isothermal for 30 min without any nitrogen gas supply. After the deposition, the heater was switched off to induce a cooling down period, while the uniformed thin layer was deposited on the Si + Au substrate.

### Surface modification of nanostructured ZnO urchins with PVDF-HFP

In order to cover the surface of ZnO urchin with PVDF-HFP, samples were dipped into a solution of 17 % poly(vinylidene fluoride-co-hexafluoropropylene) in DMF for 3 s and then dried at 30 °C for 24 h.

### Characterization of Nanostructured ZnO Urchins

The morphology of ZnO nanostructures was inspected with the Hitachi S4700 field-emission scanning electron microscope (Hitachi, Tokyo, Japan). The crystal structure properties of the nanostructures were obtained by hard X-ray low-angle reflectivity measurements, using the Philips PW1710 powder diffractometer (Philips, Amsterdam, Netherlands) with a copper anode source (Cu-Kalpha, lambda = 0.154 nm), operating at 0.8 kW and with an accuracy of 0.015° 2 theta. The intensity was detected with a proportional Xe-gas-filled detector. In order to observe the changes in the surface morphology between the uncoated (Si + Au + ZnO urchin2) and the polymer-coated (Si + Au + ZnO urchin2 + PVDF-HFP) ZnO urchins, the surface morphology of the main substrates were analysed by the atomic force microscopy (AFM)-based methodology. Changes in the surface morphologies were examined by AFM (Solver PRO, NT-MDT, Russia) in tapping mode in air. The samples were scanned with the standard Si cantilever at a constant force of 22 N/m and a resonance frequency of 325 kHz (10 nm tip radius, 95 μm tip length). The morphology was studied on 5 × 5 μm^2^, and the average surface roughness (Ra) was calculated from five representative images. Furthermore, Raman spectra of the Si + Au + ZnO urchin2 and Si + Au + ZnO urchin2 + PVDF-HFP films were recorded at room temperature using confocal Raman imaging system alpha300 R (WITec) with frequency doubled Nd: YAG laser (532 nm) in backscattered geometry (see also Additional file 1: Supplementary Materials). The power of the output laser beam at the sample was estimated to be 6 mW. Spectrometer with a grating of 1800 lines mm^−1^ was used. The acquisition time for a single spectrum was 1 min. Mapping was done for each sample on several 40 mm × 40 mm regions, each one containing 400 points with an acquisition time of 5 s for each point (result presented in the Additional file 1: Figure S2).

### Cell Culture

Madin-Darby canine kidney cells (MDCK) were cultured in the Dulbecco’s Modified Eagle’s Medium (Sigma-Aldrich, Steinheim, Germany) and Ham’s F-12 Nutrient Mixture (Sigma-Aldrich, Steinheim, Germany) (50:50, *v*/*v*) supplemented with 0.1 % penicillin/streptomycin (Gibco, Life technologies, Thermo Fisher Scientific Inc), 2.5 % foetal bovine serum (Sigma-Aldrich, Steinheim, Germany) and 4 mM Glutamax (Gibco, Life Technologies, Thermo Fisher Scientific, Inc.) in a humidified atmosphere of 5 % CO_2_/95 % air at 37 °C. The cells were routinely sub-cultured three times a week in a ratio of 1:5. Before harvesting with 1× TrypLE Select (Gibco, Life Technologies, Thermo Fisher Scientific, Inc.) for 15 min at 37 °C, the cells were washed three times with phosphate-buffered saline (PBS) without Ca^2+^ and Mg^2+^ (Sigma-Aldrich, Steinheim, Germany). After harvesting, the cells were resuspended in the medium, centrifuged at 200*×g* for 5 min and plated at a seeding density of 5 × 10^4^ cells per cm^2^ of the tissue culture 12-well plates (Sigma-Aldrich, TPP^*®*^, Steinheim, Germany) containing substrate (Si, Si + Au, Si + Au + ZnO urchin2, Si + Au + PVDF-HFP, Si + Au + ZnO urchin2 + PVDF-HFP and a plain Zn disc). All substrates were cleaned with 70 % ethanol (EtOH; Merck KGaA, Darmstadt, Germany) and autoclaved prior to use. The Si and Si + Au substrates were chosen as reference substrate to examine cell growth on the ZnO urchins and the ZnO urchins coated with PVDF-HFP surfaces.

### Cell Imaging

The cells were grown on the selected substrate for 1 week (37 °C, 5 % CO_2_). Prior to fixation, cell growth and viability were visually inspected by inverted phase-contrast light microscopy (Zeiss Axiovert A1, Carl Zeiss, Jena, Germany). After 1 week, the exposure time having been selected based on our previously published study with a similar experimental setup [27], the cells were washed with PBS (pH 7.4) and fixed for 2 h at room temperature, using a modified Karnovsky fixative, composed of 2.5 % glutaraldehyde (SPI Supplies, West Chester, PA, USA) and 0.4 % paraformaldehyde (Merck KGaA, Darmstadt, Germany) in a 1 M Na-phosphate buffer (NaH_2_PO_4_·2H_2_O and Na_2_HPO_4_·2H_2_O; all from Merck KGaA, Darmstadt, Germany). After washing them in the 1 M Na-phosphate buffer (3 × 10 min), the post-fixation of the samples was undertaken with 1 % OsO_4_ (SPI Supplies, West Chester, PA, USA; 1 × 60 min). The samples were dehydrated with a series of EtOH and acetone (absolute EtOH, acetone, Merck KGaA, Darmstadt, Germany), each step lasting 10 min. The samples were dried with hexamethyldisilazane (HMDS, SPI Supplies, West Chester, PA, USA), which was left to evaporate for 24 h before placing the samples on the aluminium holders. The ultrastructural status of the MDCK cells growing on the ZnO nanostructures’ surfaces as well as the Si and Si + Au surfaces was analysed using SEM JSM-7600 F (JEOL, Tokyo, Japan). An EDSX analysis was performed on selected regions of the samples (*n* = 2 to 4 samples in each tested group).

### Viability Assay

The cells were seeded into 12-well cell culture plates containing the selected substrate at a seeding density of 2 × 10^4^ cells per cm^2^ (1.5 mL into each well; surface area of a well is 3.7 cm^2^) and incubated in a controlled atmosphere for either 24 h or for 7 days. A neutral red uptake (NRU) assay was carried out according to the protocol published in Nature Protocols [28]. Briefly, the wells with cells on the substrate were washed three times with PBS, whereupon the substrate was removed into new, clean wells. The cells growing around the substrates remained attached to the surrounding wells; therefore, cell viability of both the old wells (with cells attached to the plastic) and the new wells (with cells attached to the substrate) was assessed using the NRU assay. Then, 1 mL of the freshly prepared medium, supplemented with 40 μg/mL neutral red (NR), was added to each well of both the cell culture plates. After 2 h of incubation at 37 °C, the medium was removed and the NR dye, trapped inside the cellular lysosomes, was released by the addition of 0.4 mL of de-staining solution (50 % of EtOH, 1 % of glacial acetic acid and 49 % of deionized H_2_O; *v/v* ) per well. For the quantification, 0.1 mL of the sample were transferred into a 96-well plate and the fluorescence of NR (ex: 530 nm; em: 645 nm) was measured. The cell viability of the cells grown on the control substrate (cover glass), the reference group substrates (Si, Si + Au, Si + Au + PVDF-HFP) and finally the main group substrates (Si + Au + ZnO, Si + Au + ZnO + PVDF-HFP) was assessed. Additionally, the cell viability of cells grown on plain Zn discs was tested in order to assess the viability of the cells grown on a plain, untreated Zn surface. All cell viability tests were performed following the same experimental procedure (NRU assay).

### Quantification of the Zn Content of the Cell Medium

During the NRU assay, the Zn content of the cell medium was quantified to check the level of ZnO dissolution in the cell media. In order to assess the release of Zn within the first half of the 7-day exposure, the cell medium was sampled after 3 days of cell exposure, then the cell medium was replaced with a fresh one and again sampled after another 4 days (at the end of the exposure). The selection of the sampling time was in line with the cell viability assessment (NRU assay). Prior to the analysis, 0.75 mL of concentrated nitric acid (65 % HNO_3_, *pro analysis*, Carlo Erba Reagenti, Arese, Italy) was added to 0.75 mL of the cell medium from each of the test vessels. The samples were digested in the Milestone Ethos E (Bergamo, Italy) microwave lab station equipped with an SK-10 high-pressure segmented rotor and 3 mL quartz microsampling inserts. The digestion was conducted at 180 °C and 600 W power, with step 1 (heating) lasting 15 min, step 2 (constant temperature) lasting 10 min, and cooling to 60 °C lasting 45 min. The total Zn concentrations in the samples were measured by flame atomic absorption spectroscopy (AAS; Perkin-Elmer AAnalyst 100, Waltham, MA, USA).

## Results and Discussion

### Morphological and Crystalline Properties of the ZnO Urchin

**Fig. 1:**
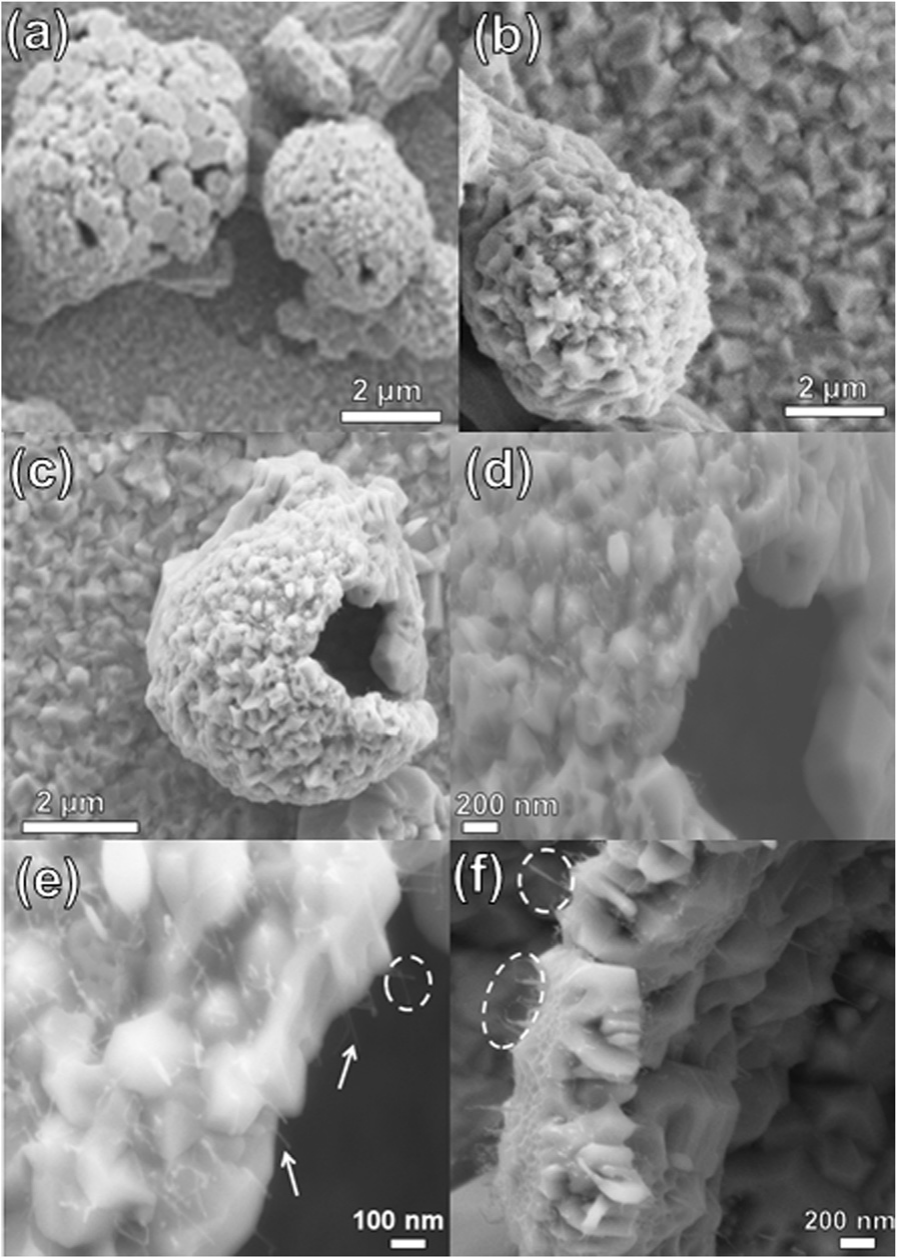
**a**–**f** SEM images of a hollow ZnO microsphere grown on the Si + Au substrate. The weight ratio of the source material was 1:1:0 (C:ZnO:SnO_2_)

Figure 1 shows the SEM images of a hollow ZnO microsphere grown on the Si + Au substrate, where the weight ratio of the source materials was 1:1:0 (C:ZnO:SnO_2_). The images show that the whole substrate was covered with hexagonal discs (polyhedral crystal). Textured microspheres with small open mouths were also formed and were loosely bound to the substrate. The top view of the sample shows that the hollow ZnO microsphere is made up of numerous hexagonal discs (Fig. 1a–f). The hexagonal discs’ diameter is in the range of 100–150 nm, with a thickness of about 50 nm. The numerous discs are assembled to form hollow textured microspheres. Figure 1c, d shows the obtained hollow ZnO microsphere, where the cavities of the sphere can be clearly seen. The side wall of the hollow microsphere exhibited in the Fig. 1e, f, displaying numerous 1D NWs with a low density starting a growth pointing toward the centre of the polyhedral crystal.

**Fig. 2:**
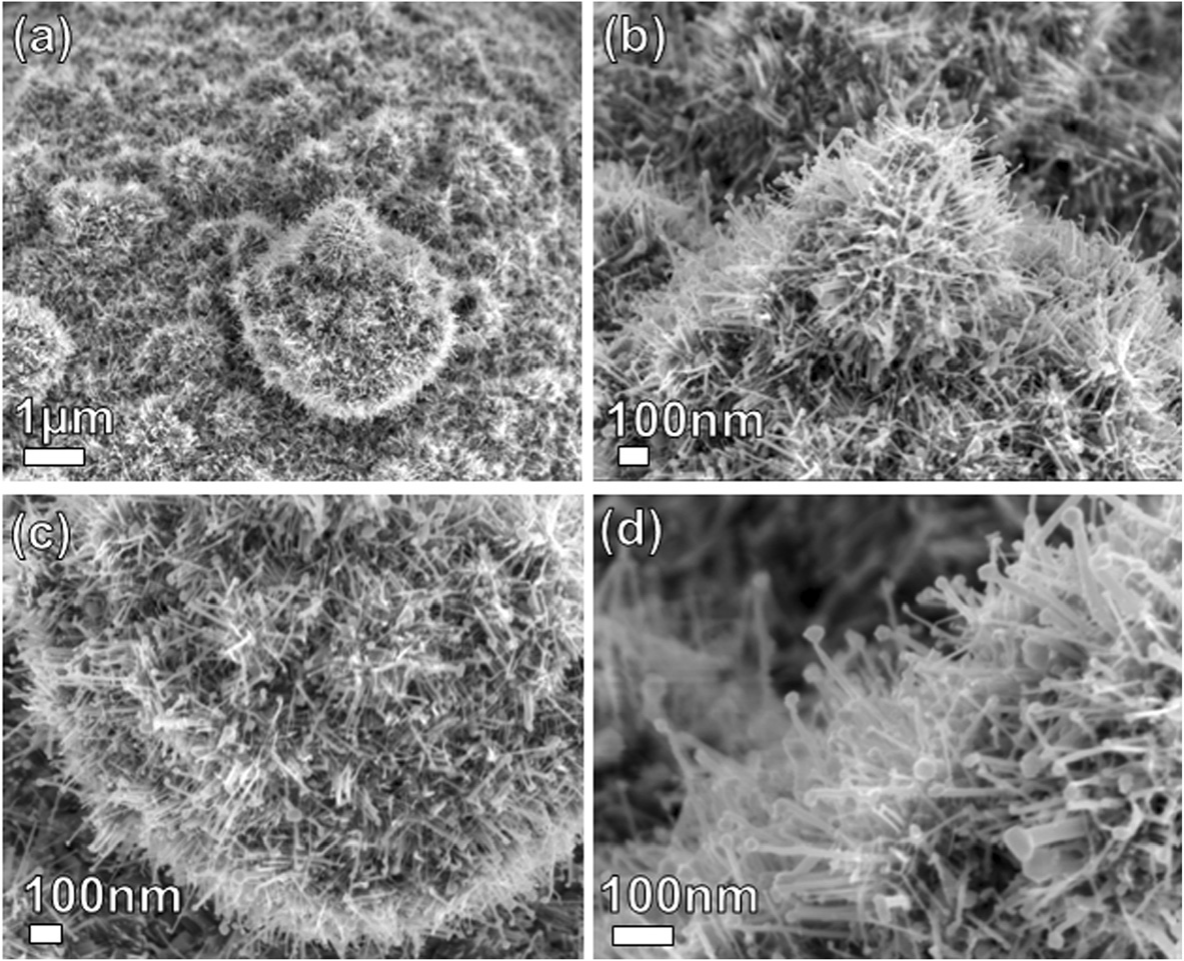
**a**–**d** SEM images of a hollow ZnO urchin1 grown on the Si + Au substrate. The weight ratio of the source materials was 2:1:1 (C:ZnO:SnO_2_)

**Fig. 3:**
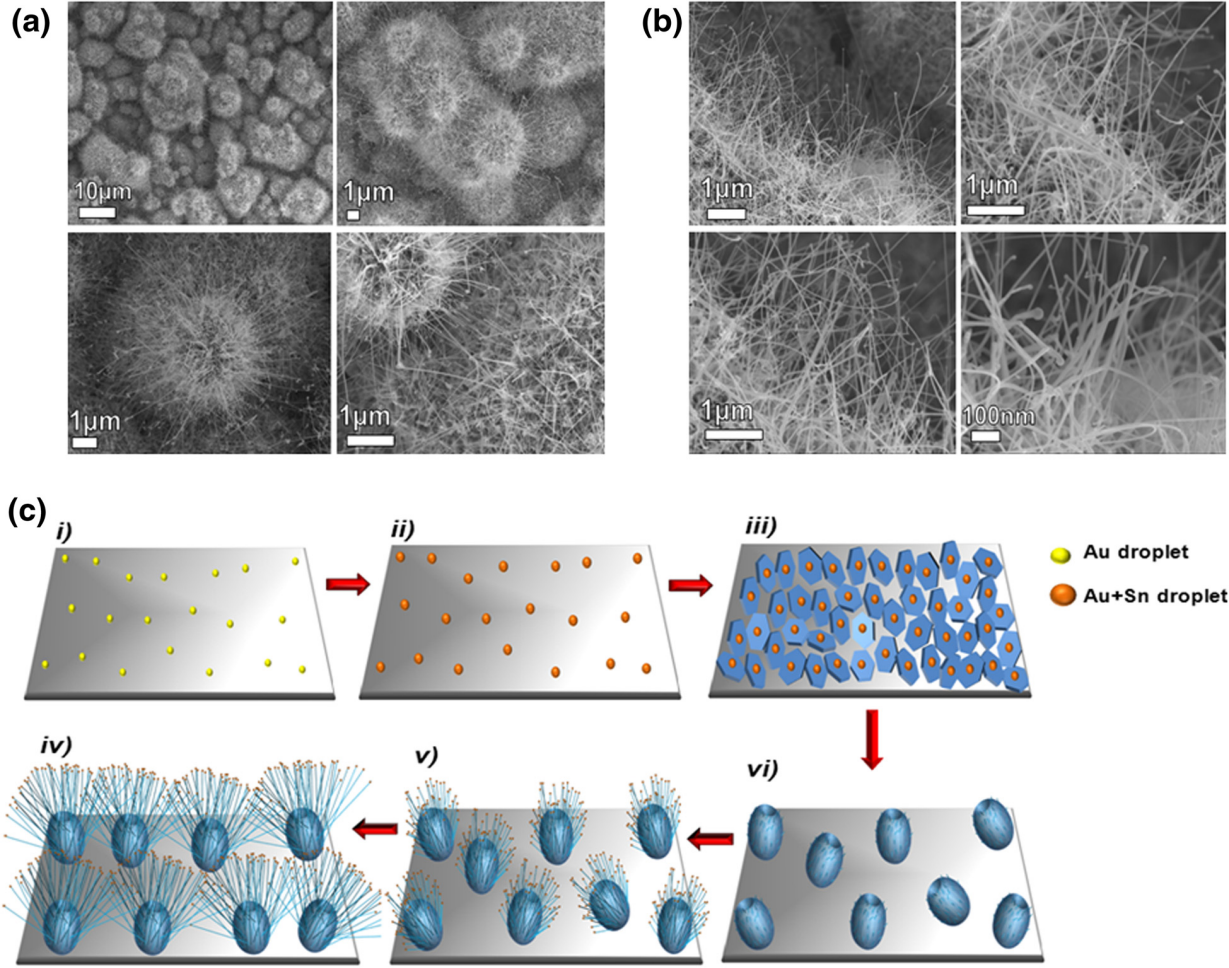
SEM images and a shematic presentation of ZnO nanostructures grown on the substrates. **a** SEM images of ZnO urchin2. **b** SEM images of ZnO NWs grown on a hollow sphere (urchin’s acicula). The weight ratio of source materials was 3:2:1(C:ZnO:SnO_2_). **c** A schematic illustration of the formation of hollow ZnO urchins: the process involves the formation of *i*) Au and *ii*) Au + Sn catalyst nano-droplets; *iii*) polyhedral Zn discs on a silicon substrate; *iv*) the formation of spheres from the Zn discs, an oxidation of the spheres to form a thin layer of ZnO; *v*) the sprouting of small ZnO NWs, a continuous evacuation of the Zn core; and finally *vi*) the growth into hollow ZnO urchin2

Figure 2 shows SEM images of ZnO nanostructures grown on the Si + Au substrate, where the weight ratio of the source materials was 2:1:1 (C:ZnO:SnO_2_). At this deposition, 1D NWs with a high density are radially aligned on the surface of the microspheres, and the nanowire branches have particles at their growing ends (the presence of particles at the tips of nanowires is suggestive of a vapor-liquid-solid (VLS) growth process). These novel structures are named “hollow ZnO urchins1” due to their resemblance to the naturally occurring sea urchin. Figure 2d shows acicula crystallites radiating from the “urchin” centre with a uniform size distribution. The ZnO NWs possess diameters and lengths of about 50 and 200 nm, respectively. As opposed to the hollow ZnO urchin1, when the weight ratio of the source materials was 3:2:1 (C:ZnO: SnO_2_), the thinner and longer NWs were radially aligned on the surface of the microsphere, forming a structure named “hollow ZnO urchin2”. SEM images of hollow ZnO urchin2 are presented in Fig. 3. The novel phenomenon in this structure is the contact between the acicula of the adjacent urchins, which is formed due to long acicula crystallites radiating from the “urchin’s” centre (Fig. 3a).

Figure 3b shows high-magnification SEM images of NWs grown on the surface of hollow microspheres. The images clearly show the presence of particles at the tips of NWs, which is suggestive of a VLS growth process [29, 30]. The diameter and length of NWs in this structure are around 20 nm and 5 μm, respectively. In the VLS process, the source materials have a critical role in the growth of ZnO nanowires. For this reason, we have also investigated in the current study the influence of the source materials ratio (ZnO:SnO_2_) on the morphology of ZnO nanostructures. In order to elucidate the role of source materials in the growth of the ZnO urchins and branched ZnO NWs, the structures obtained at the different ZnO:SnO_2_ ratios are discussed and compared together in the following text.

During the first deposition, when the ZnO:SnO_2_ ratio was 1:0 and Au was the single element in the catalyst droplet, only very thin and small wires were observed to be growing on the surface of the hollow spheres (ZnO microsphere) [31]. In the other deposition, with the introduction of SnO_2_ to the source materials (ZnO:SnO_2_ ratio was 1:1), short and thick NWs with a high density were radially aligned on the surfaces of the microspheres (ZnO urchins1) [32]. Finally, when the ratio of ZnO:SnO_2_ was 2:1, thin and long NWs were radially aligned on the surface of the microspheres (ZnO urchins2). The NWs grown on the ZnO urchins1 were shorter and thicker than the nanowires grown on the ZnO urchins2 due to lower Zn vapour pressure and larger Au + Sn catalyst nuclei, whereas on the ZnO urchins2, the nanowires were longer and thinner [33]. Thin and long NWs of ZnO urchins2 are a consequence of the abundant Zn vapour surrounding the substrate and smaller Au + Sn catalyst nuclei, which lead to a fast initiation of the growth of longer and thinner NWs [31, 34]. In general, we can presume that the growth of hollow ZnO urchins included Zn powder vaporization; the solidification of liquid droplets forming a Zn polyhedral disc, surface oxidation, Zn sublimation; and the catalytic-assisted growth of one-dimensional NWs. The growth mechanism of the ZnO urchins is presented in the Fig. 3c. Additional discussion on the growth mechanism of the ZnO urchins is available in the Additional file 1.

**Fig. 4:**
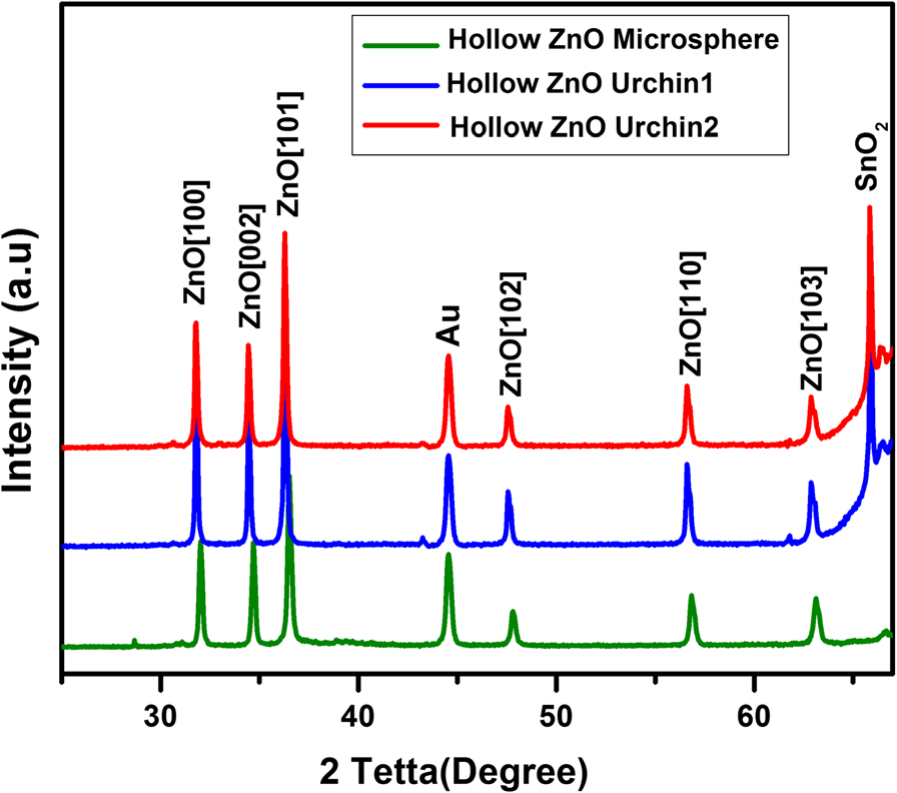
XRD pattern of the ZnO nanostructures grown at different depositions

The X-ray diffraction (XRD) pattern of the nanostructures grown at different depositions, namely the hollow ZnO microsphere (ZnO:SnO_2_→1:0), the hollow ZnO urchin1 (ZnO:SnO_2_→1:1), and the hollow ZnO urchin2 (ZnO:SnO_2_→2:1), is shown in Fig. 4. The ZnO diffraction peaks of all samples correspond well with the (100), (002), (101), (102), (110), and (103) planes of the standard pattern of the hexagonal wurtzite phase of ZnO with a lattice constant of a = b = 0.3249 nm and c = 0.5205 nm, (space group: P63mc, JPCPDF file no. 36-1451). The Au peak at 44.4 corresponds with the (200) plan, where the contributions of gold NPs as catalysts for all three samples are considered. The SnO_2_ peak at 65.8, which corresponds with the (301) plane, is responsible for the SnO_2_ contribution in the catalyst droplet for the hollow ZnO urchin1 and the hollow ZnO urchin2 [31]. For the cell culture experiments, the ZnO urchin2, which possesses the longer and thinner NWs, were selected.

**Fig. 5:**
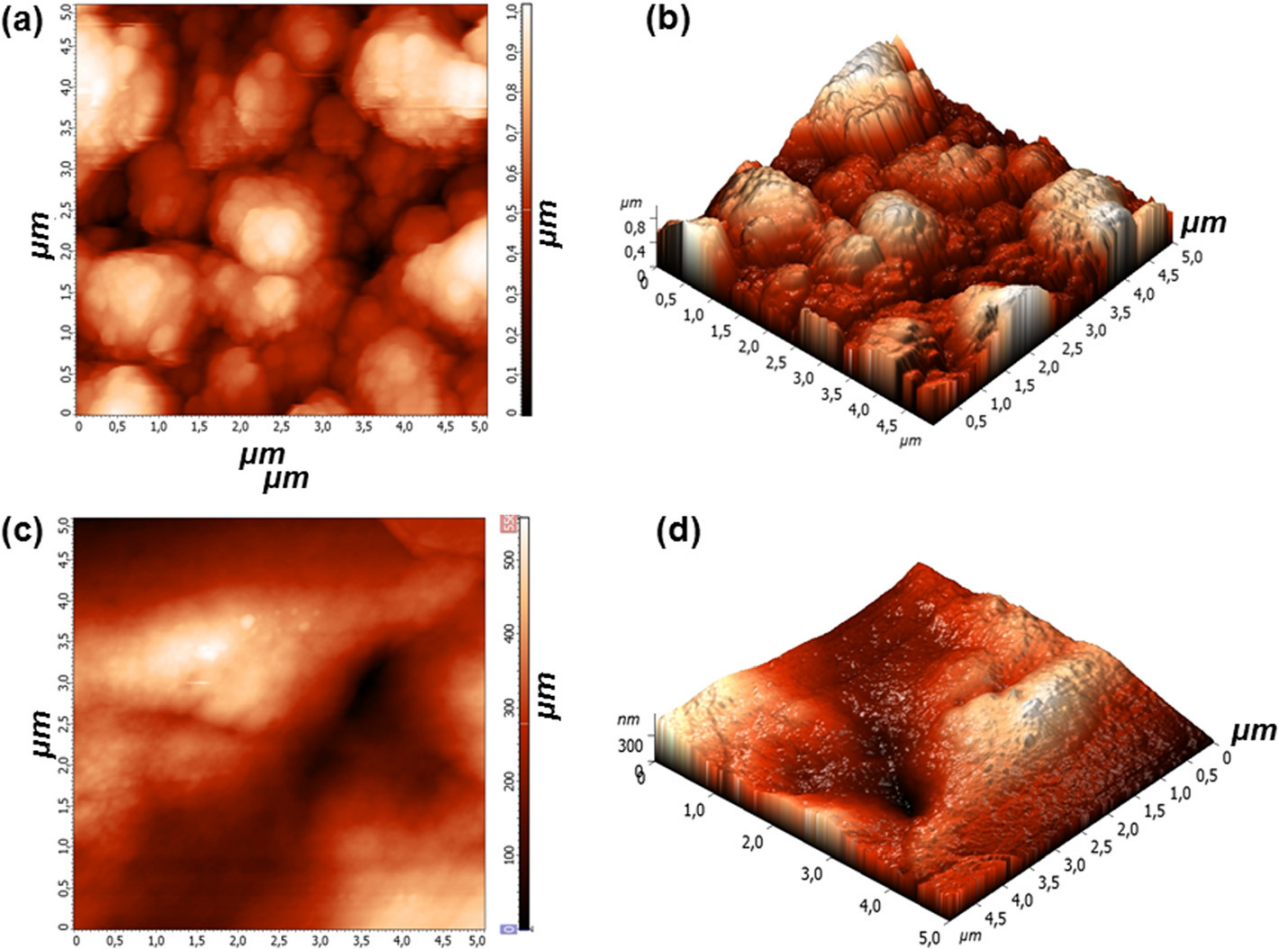
Surface morphology of substrate obtained by AFM. **a** Si + Au + ZnO urchin2 height. **b** Si + Au + ZnO urchin2 corresponding 3D image. **c** Si + Au + ZnO urchin2 + PVDF-HFP height. **d** Si + Au + ZnO urchin2 + PVDF-HFP corresponding 3D image

Changes in the surface morphology between the uncoated (Si + Au + ZnO urchin2) and the polymer-coated (Si + Au + ZnO urchin2 + PVDF-HFP) surfaces were analysed by AFM, and the results are presented in Fig. 5. The height and the corresponding 3D image of the Si + Au + ZnO urchin2 surface are given in Fig. 5a, b and in Fig. 5c, d for the Si + Au + ZnO urchin2 + PVDF-HFP. Both samples show clearly visible differences in the surface morphology. The uncoated sample in Fig. 5b exhibits microsphere structure (as already shown by the SEM analysis in the Figs. 1, 2 and 3), and the measured average surface roughness was about 165.9 nm. However, in the case of the polymer-coated surface, the morphology was quite dissimilar: there are no microspheres visible on the surface and the average surface roughness is significantly lower, about 83 nm. This information is highly valuable as the differences in the surface morphology could to some extent influence the biological response of the MDCK cells, as it is discussed later. In agreement with the AFM results, the Raman spectra of the Si + Au + ZnO urchin2 and Si + Au + ZnO urchin2 + PVDF-HFP (Additional file 1: Figure S2) are also distinctly different.

### Cell Growth and Viability

**Fig. 6:**
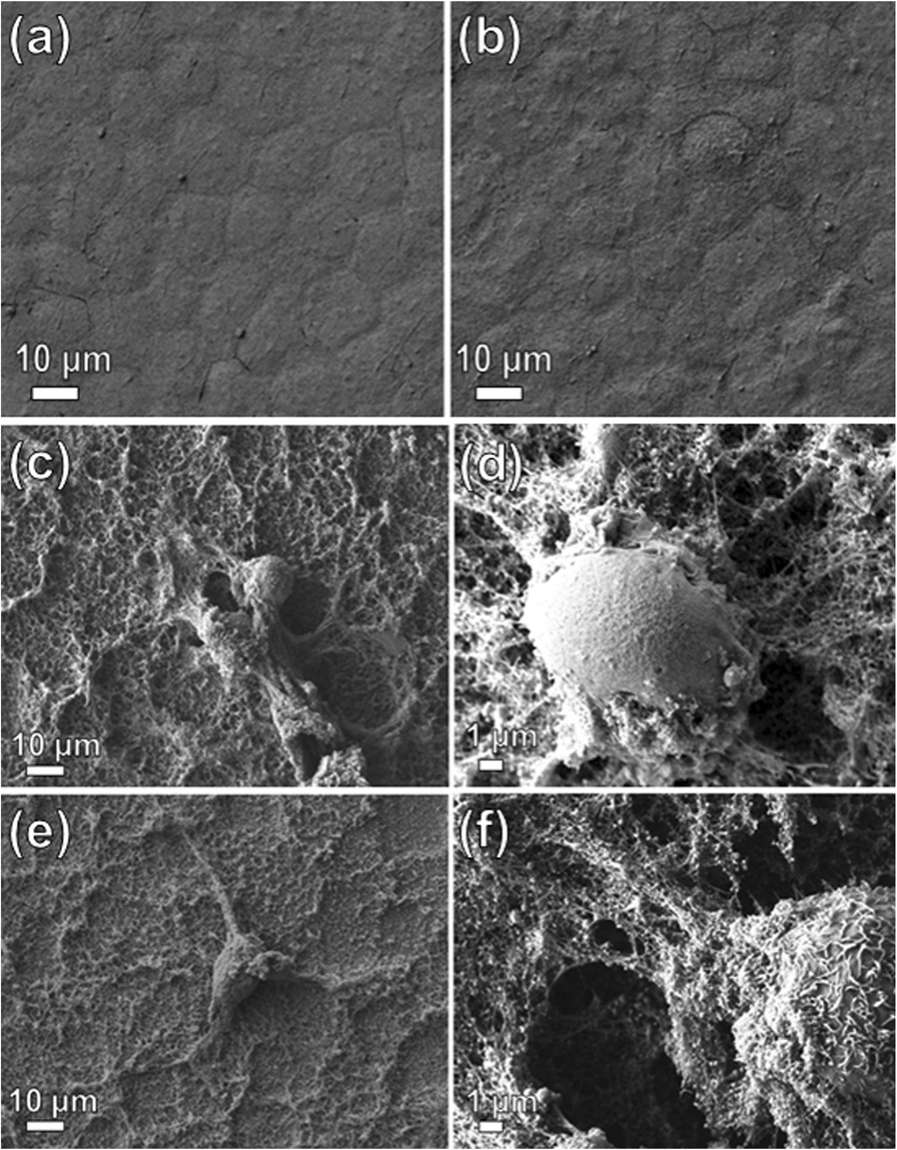
SEM images of MDCK cells after 7-day growth on the substrates. The different substrates presented the following: **a** Si, **b** Si + Au, and **c**–**f** Si + Au + ZnO urchin2. **a**, **b** A confluent layer of cells was formed on the surface of both the reference materials (cell growth was not affected). **c**–**f** Individual cells rather than a confluent layer are present on the Si + Au + ZnO urchin 2 nanostructured substrate. Note: A layer of proteins (either from cell culture medium, or from cell debris) is formed around the individually attached cells

**Fig. 7:**
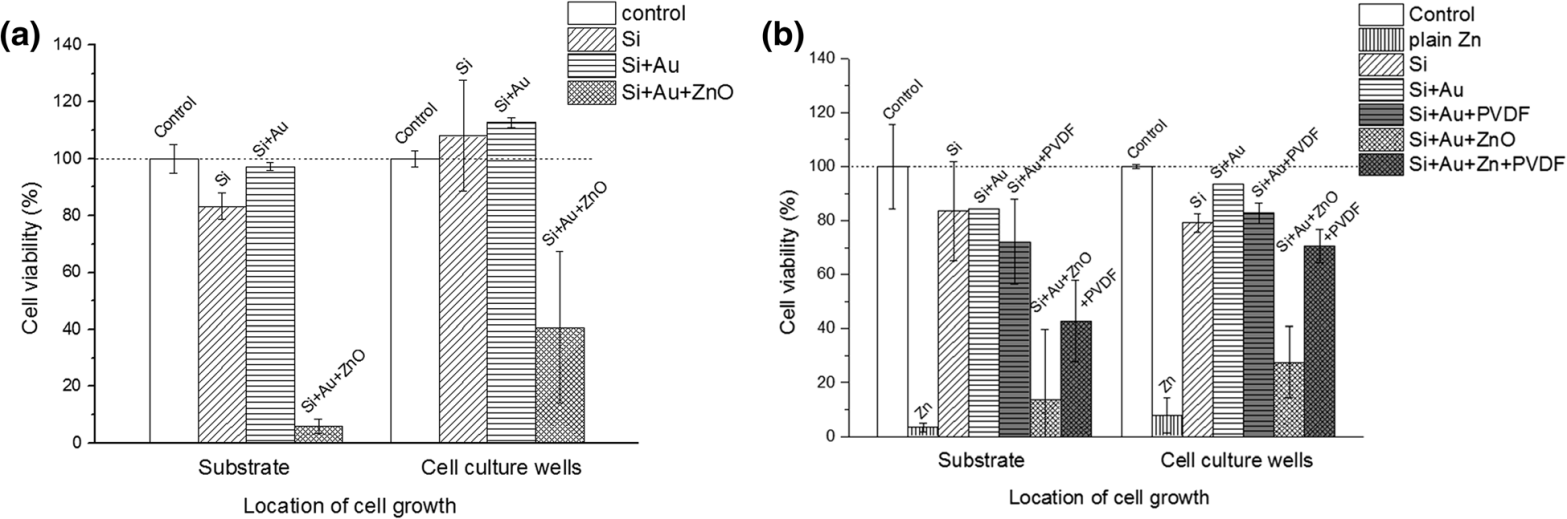
Cell viability of MDCK after (a) 7-day and (b) 24-h growth on the surfaces. Cells were grown both on the substrate (*left columns*) and on the same cell culture wells around the substrate’ position (*right columns*), either without PVDF-HFP coverage (**a**), and substrates with or without PVDF-HFP coverage (**b**). The viability of cells was estimated on the basis of the fluorescence values obtained by the NRU assay, normalized to the viability of the control group, i.e. the cells grown on cover glass (location of cell growth: substrate) or around it (cell culture wells). Analysis of each column was according three to four replicates

The cytotoxicity effect can be manifested as a decreased number of attached cells and an increased number of floating cells or by a decreased NR fluorescence (expressed as percent of cell viability in Fig. 7). Fig. 6 shows SEM images of MDCK cells after 1 week of growth on the (a) Si, (b) Si + Au, and (c–f) Si + Au + ZnO urchin2 surface. The confluent layer of viable cells was formed on both the reference surfaces, Si and Si + Au (Fig. 6a, b). On the other hand, the cell growth on the Si + Au + ZnO urchin2 surface was not confluent, since only individual cells were present; moreover, the few cells that remained on the ZnO urchin2 surface were mostly not viable (roundly shaped; Fig. 6c–f). The cytotoxic effect of the Si + Au + ZnO urchin2 surface, as assessed by the NRU assay, was present already after 24 h and 7 days of exposure (Fig. 7a, b). Viability result presented in Fig. 7 proved that the presence of the ZnO urchin2 on the substrate significantly decreased the viability of cells, independently of the direct or indirect cell contact with the ZnO urchin2, both after 7 days (Fig. 7a) and 24 h (Fig. 7b) of exposure. The cell viability was reduced both when the cells were attached to the surfaces, as well as when they were growing on the surrounding surfaces in the same wells. The carrier substrate of ZnO urchins2 (i.e. the reference substrate: Si and Si + Au) had no effect on the viability, neither on the cells attached to the platelets nor to the cells that were grown in the same wells containing the Si or Si + Au surfaces (Fig. 7a, b).

Furthermore, the effect of the PVDF-HFP coating was tested in order to reveal the impact of polymer coating on the ZnO nanostructure cytotoxicity (Fig. 7b). The PVDF-HFP coating significantly increased the cell viability of the cells grown on the ZnO urchin2 surfaces and the cells growing on the surrounding cell culture dish (Fig. 7b) in comparison to the same surfaces without the PVDF-HFP coating. We hypothesized that the presence of dissolved Zn in the wells induced cell death of the surrounding cells. Similar results were obtained by other researchers [35] with different cell lines (fibroblasts, umbilical vein endothelial cells, capillary endothelial cells). They observed the inability of cells to adhere and subsequently spread on surfaces with ZnO nanorods, suggesting that the lack of initial spreading is probably the main cause of cell death rather than the long-term exposure to ZnO. In contrast, we have found that the ZnO NW-coated material (ZnO urchin2) significantly reduced the growth and viability of cells present in the same well of cell culture dish already after a 24-h exposure (Fig. 7b). Therefore, we explain cell death by the known phenomenon of Zn dissolution in the aqueous media. Several groups [21, 36, 37] have reported on the strong Zn dissolution from ZnO NWs into the cell culture media, followed by Zn internalization, which has been shown to induce cytotoxicity. Recently, a few research groups measured the amount of dissolved Zn in the cell culture media upon subjection to ZnO substrates with different nanotopography and the level of dissolved Zn corresponded with the inverse trend of the adherent cell (macrophage) viability [38–40].

### Zn Dissolution in the Cell Media

Information about the Zn quantity in the cell media is presented in Table 1. According to gathered information, the Zn content in the cell culture media sampled from the control group and from the wells where the cells were exposed only to the reference substrate (Si and Si + Au) was approximately equal (Table 1). Therefore, these values denote the background Zn content in the cell medium with possible (but negligible) contribution of the Zn traces present in the reference substrate (Si or Si + Au) or in the cell culture dish. However, the Zn content in the cell culture media from the wells where the cells were grown on the ZnO urchin2 was more than five levels of magnitude higher, both after 3 days and after the additional 4 days of the NRU assay (Table 1). We assume that the amount of released Zn was proportional to the exposure time [20]. The PVDF-HFP coating reduced the Zn release by approximately 50 %. The release of Zn was higher from the plain Zn substrate in comparison to the ZnO urchin2 substrate (Table 1). The obtained results have shown that the thin layer of PVDF-HFP decreased the Zn dissolution rate and therefore reduced the cytotoxicity of the substrate.

**Table 1:** The release of Zn from the different cell growth substrates into the cell medium

Cell growth substrate type	Total Zn concentration in the cell medium at different sampling times (mg/L, mean ± SD)		
	3 days (experiment 1)	7 days (experiment 1)	16 h (experiment 2)
Cell medium (background)	0.08 ± 0.02 (*n* = 4)	0.09 ± 0.01 (*n* = 4)	0.09 ± 0.01 (*n* = 4)
Glass cover slip (negative control)	0.12 ± 0.06 (*n* = 4)	0.08 ± 0.02 (*n* = 4)	0.18 ± 0.04 (*n* = 4)
Si	0.05 ± 0.01 (*n* = 4)	0.04 ± 0.02 (*n* = 4)	0.16 ± 0.09 (*n* = 3)
Si + Au	0.07 ± 0.04 (*n* = 4)	0.04 ± 0.01 (*n* = 4)	0.18 ± 0.11 (*n* = 3)
Si + Au + ZnO urchin2	16.4 ± 2.0 (*n* = 3)	23.4 ± 2.5 (*n* = 3)	13.8 ± 3.9 (*n* = 3)
Si + Au + ZnO urchin2 + PVDF-HFP	NA	NA	6.9 ± 1.5 (*n* = 2)
Plain Zn disc (positive control)	NA	NA	25.4 ± 5.3 (*n* = 4)

There were no cells in the negative control to account for the Zn content in the cell medium and in the culture wells

*Abbreviations: NA* data not available, *SD* standard deviation, *n* number of analysed data

## Conclusions

For the first time, we synthesized the new nature-inspired ZnO urchins on the Si substrate with the traditional carbothermal method. The length of the acquired new nanostructures in the urchin’s acicula is controllable according to the source material ratio. The biological experiments indicated that the ZnO urchins possess a high degree of cytotoxicity which occurs due to its instability in the cell medium and the shedding of a high amount of Zn to the cell media. To overcome this problem, we coated the surface of the ZnO urchins with PVDF-HFP. The surface coating of the ZnO urchins with PVDF-HFP significantly decreased the cytotoxicity of the ZnO urchins as well as its Zn shedding, but still, a problem remains regarding the stability of PVDF-HFP on the surface of the ZnO urchins. Further research should focus on the ZnO urchin surface modification, combined with other methods to grant access to a more stable coverage, including other different types of biocompatible polymers.

## Additional file

Additional file 1:
**Growth mechanism of the ZnO urchin.** (DOCX 626 KB)

## Abbreviations

EtOH: ethanol
MDCK: Madin-Darby canine kidney
NGs: nanogenerators
NPs: nanoparticles
NR: neutral red
NRU: neutral red uptake
NWs: nanowires
PVDF-HFP: poly(vinylidene fluorid-co-hexafluoropropylene)
SEM: scanning electron microscopy
ZnO: zinc oxide
