# Supplementary material for: Growth of a Novel Nanostructured ZnO Urchin: Control of Cytotoxicity and Dissolution of the ZnO Urchin
Source: Nanoscale Res Lett. 2015 Nov 16;10:441. doi: 10.1186/s11671-015-1145-3 (PMC4646880; doi:10.1186/s11671-015-1145-3)
Supplement: Additional file 1: — Growth mechanism of the ZnO urchin. (DOCX 626 KB) [file 11671_2015_1145_MOESM1_ESM.docx]

# SUPPLEMENTARY MATERIALS to the manuscript “*Growth of a novel nanostructured ZnO urchin: control of cytotoxicity and dissolution of the ZnO urchin*” (by R. Imani, B. Drašler, V. Kononenko, T. Romih, K. Eleršič, J. Jelenc, I. Junkar, M. [Remškar](https://www.google.si/url?sa=t&rct=j&q=&esrc=s&source=web&cd=1&cad=rja&uact=8&ved=0CCAQFjAA&url=http%3A%2F%2Fwww-f5.ijs.si%2Findex.php%3Flang%3Dsl%26i%3D91%26p%3Dosd&ei=T6vtVIrhHoTaaKSfgsAC&usg=AFQjCNHwLmZODbW9NRk2QFdsxy1fQdozlw&sig2=15BgkL7c5F7tjKI2hn-CBw&bvm=bv.86956481,d.d2s), D. Drobne, V. Kralj-Iglič, A. Iglič)

**Growth Mechanism of the ZnO Urchin**

To find common characteristics of the possible growth mechanism of hollow ZnO urchins, we inspevted different ZnO urchin shapes using Scanning Electron Microscopy (SEM), as presented in the Figure S1. Graphite was introduced into the raw materials in order to reduce the oxides into metals, as proposed by [1]:

2ZnO (s) + C (s) → 2Zn (g) + CO_2_ (g) (1)

SnO_2_ (s) + C (s) → SnO (g) + CO (g) (2)

SnO_2_ (s) + C (s) → Sn (g) + CO_2_ (g) (3)


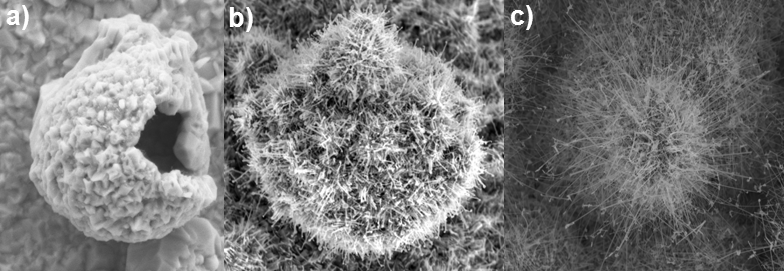


**Figure S1.** SEM images of a) a hollow sphere, b) a hollow ZnO urchin with short acicula, c) a hollow ZnO urchin with long acicula.

The generated Sn vapor initiated a catalyst droplet-assisted growth in the early stage of the VLS process. According to the alloy phase diagram of Au and Sn, at the temperature of ∼282 °C, Au and Sn formed a eutectic Au–Sn alloy. The Au–Sn droplet surface has a higher sticking coefficient and is therefore the preferred absorption site for incoming Zn vapor. The sublimated Zn atoms were carried downstream by nitrogen carrier gas. In the lower temperature region, the Zn atoms were chemically adsorbed on the surface of the Au+Sn droplet. The liquid droplet quickly solidified on the substrate and formed a Zn polyhedral crystal; the residual oxygen in the growth chamber was then able to oxidize the surface of the Zn polyhedral. During the formation of the polyhedral Zn disk, the Au+Sn catalyst remained at its front. The presence of the Au+Sn catalyst on the surface of the polyhedral ZnO disk is beneficial to the preferred aligned growth of ZnO nanowires. The polyhedral Zn disks function as building blocks for the formation of the Zn spheres. The surface of the Zn spheres can be easily oxidized by the residual oxygen in the system and forms a thin ZnO layer on the Zn sphere surface. Since Zn has a lower melting point (419 °C) than the thin layer of the naturally oxidized ZnO shell (1975 °C), the latter is quite stable.

Further thermal evaporation led to the sublimation of the Zn core. The shell collapsed and formed shell structures with a hollow core. With prolonged reaction, small ZnO nanowires sprouted out epitaxially from the surface via the VLS mechanism. The formation of the ZnO nanowires on the surface of hollow ZnO microspheres by the VLS mechanism has been proven by the presence of Au particles at the tops of the nanowires. The VLS growth of semiconductor nanostructures was originally explained by supersaturation of the liquid catalyst with the growth materials. In fact, a higher growth rate of the ZnO nanowires requires more of the impacting Zn atoms to stick to the surface of the catalyst droplet.

**Raman Spectroscopy**

Figure S2 shows Raman spectra for Si+Au+ ZnO urchin2 and Si+Au+ ZnO urchin2+PVDF-HFP, which are distinctly different. Normally, most Raman active modes of PVDF (α-phase) appear in the frequency range of 450-670 cm^-1^. In the Raman spectra of Si+Au+ZnO urchin2+PVDF-HFP (S2), peaks at 557, 576, 632 cm^-1^ can be attributed to the α-phase of PVDF [2–4].


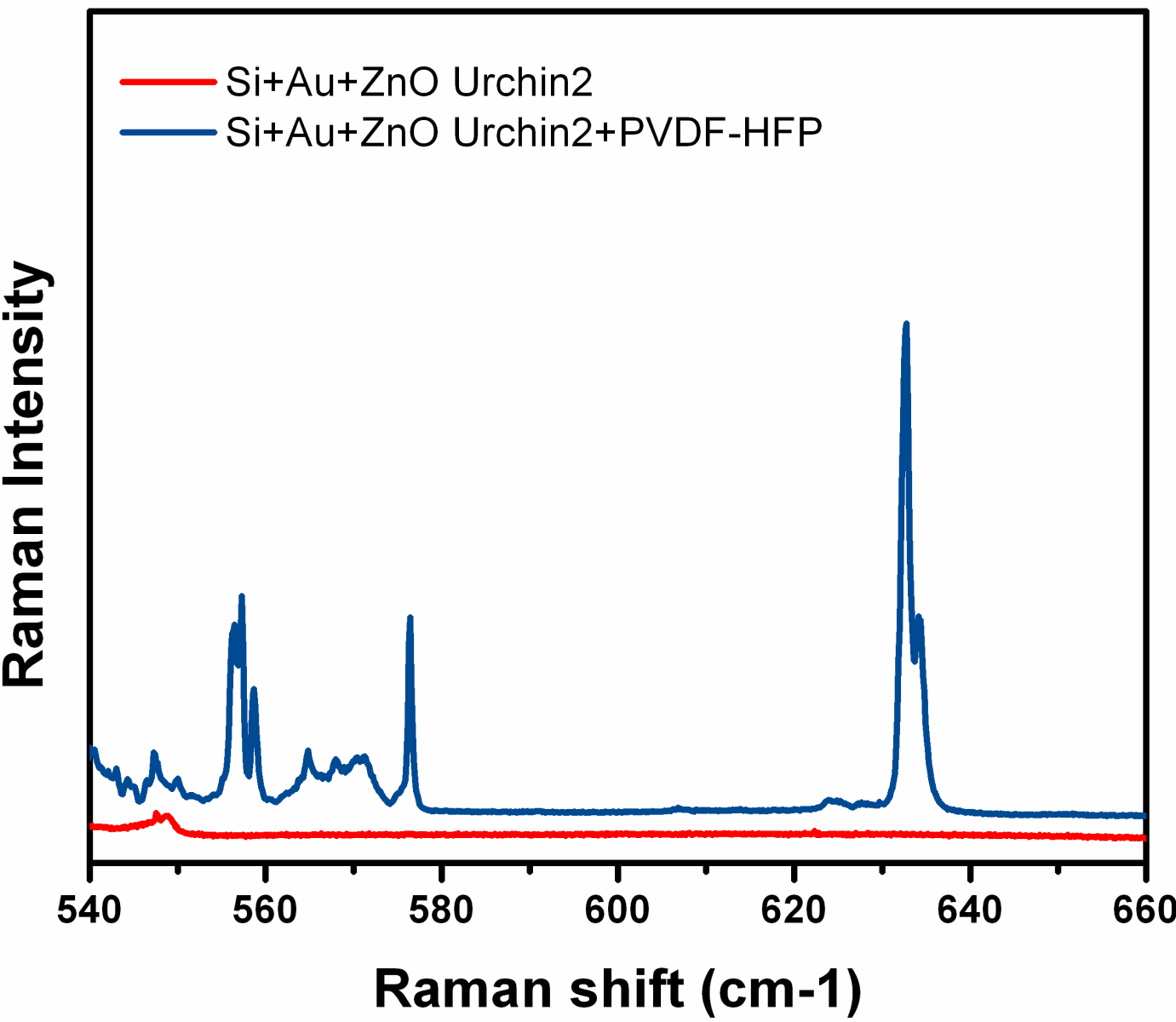


**Figure S2.** Raman spectra of the Si+Au+PVDF-HFP and Si+Au+ZnO and Si+Au+ZnO+PVDF-HFP surfaces.

**References**

1. Gao PX, Wang ZL. Mesoporous Polyhedral Cages and Shells Formed by Textured Self-Assembly of ZnO Nanocrystals. J Am Chem Soc. 2003; 125:11299–11305.

2. Yang Y, Ramalingam S, Wu G, et al. Perturbing effects of bulky comonomers on the chain conformation of poly(vinylidene fluoride). Polymer (Guildf). 2008; 49:1926–1933.

3. Ericson H, Kallio T, Lehtinen T, et al. Confocal Raman Spectroscopic Investigations of Fuel Cell Tested Sulfonated Styrene Grafted Poly(vinylidene fluoride) Membranes. J Electrochem Soc. 2002; 149:A206–A211.

4. Yang CC, Lian ZY, Lin SJ, et al. Preparation and application of PVDF-HFP composite polymer electrolytes in LiNi0.5Co0.2Mn0.3O2 lithium-polymer batteries. Electrochim Acta. 2014; 134:258–265.
